# Supplementary material for: Modeling glioblastoma heterogeneity as a dynamic network of cell states
Source: Mol Syst Biol. 2021 Sep 16;17(9):e10105. doi: 10.15252/msb.202010105 (PMC8444284; doi:10.15252/msb.202010105)
Supplement: Supplementary file 6 — Source Data for Figure 5 [file MSB-17-e10105-s004.zip › Figure5A_sourcedata/GSEA_3017/nosigdb_stateC.GseaPreranked.1622795002785/NB_KI_CLUSTER5.html]

Details for gene set NB\_KI\_CLUSTER5[GSEA]

|  || Dataset | state13017 |
| Phenotype | NoPhenotypeAvailable |
| Upregulated in class | na\_pos |
| GeneSet | NB\_KI\_CLUSTER5 |
| Enrichment Score (ES) | 0.7168141 |
| Normalized Enrichment Score (NES) | 4.137779 |
| Nominal p-value | 0.0 |
| FDR q-value | 0.0 |
| FWER p-Value | 0.0 |
Table: GSEA Results Summary

  

Fig 1: Enrichment plot: NB\_KI\_CLUSTER5      
 Profile of the Running ES Score & Positions of GeneSet Members on the Rank Ordered List

  

| PROBE | GENE SYMBOL | GENE\_TITLE | RANK IN GENE LIST | RANK METRIC SCORE | RUNNING ES | CORE ENRICHMENT || 1 | ATAD2 |  |  | 7 | 0.649 | 0.0379 | Yes |
| 2 | HIST1H4C |  |  | 9 | 0.603 | 0.0828 | Yes |
| 3 | HIST1H1C |  |  | 11 | 0.583 | 0.1263 | Yes |
| 4 | EZH2 |  |  | 14 | 0.574 | 0.1672 | Yes |
| 5 | CLSPN |  |  | 17 | 0.561 | 0.2071 | Yes |
| 6 | HMGB2 |  |  | 20 | 0.534 | 0.2450 | Yes |
| 7 | CHAF1A |  |  | 27 | 0.494 | 0.2726 | Yes |
| 8 | PCLAF |  |  | 29 | 0.484 | 0.3083 | Yes |
| 9 | UBE2T |  |  | 31 | 0.475 | 0.3434 | Yes |
| 10 | GMNN |  |  | 38 | 0.453 | 0.3678 | Yes |
| 11 | PCNA |  |  | 48 | 0.435 | 0.3856 | Yes |
| 12 | HIST1H1E |  |  | 51 | 0.430 | 0.4154 | Yes |
| 13 | BRCA1 |  |  | 54 | 0.410 | 0.4437 | Yes |
| 14 | DHFR |  |  | 55 | 0.405 | 0.4750 | Yes |
| 15 | SMC2 |  |  | 63 | 0.386 | 0.4926 | Yes |
| 16 | DEK |  |  | 74 | 0.373 | 0.5038 | Yes |
| 17 | NASP |  |  | 75 | 0.371 | 0.5325 | Yes |
| 18 | SMC4 |  |  | 78 | 0.365 | 0.5573 | Yes |
| 19 | GINS2 |  |  | 83 | 0.360 | 0.5781 | Yes |
| 20 | DUT |  |  | 87 | 0.356 | 0.6004 | Yes |
| 21 | MIS18BP1 |  |  | 89 | 0.355 | 0.6261 | Yes |
| 22 | MCM7 |  |  | 91 | 0.352 | 0.6516 | Yes |
| 23 | MCM3 |  |  | 114 | 0.324 | 0.6378 | Yes |
| 24 | HMGN2 |  |  | 126 | 0.312 | 0.6425 | Yes |
| 25 | DNMT1 |  |  | 127 | 0.311 | 0.6666 | Yes |
| 26 | FBXO5 |  |  | 130 | 0.308 | 0.6869 | Yes |
| 27 | CENPK |  |  | 139 | 0.299 | 0.6959 | Yes |
| 28 | HIST2H2AC |  |  | 161 | 0.281 | 0.6805 | Yes |
| 29 | CIP2A |  |  | 170 | 0.272 | 0.6874 | Yes |
| 30 | HELLS |  |  | 171 | 0.271 | 0.7084 | Yes |
| 31 | DDX11 |  |  | 181 | 0.265 | 0.7130 | Yes |
| 32 | CDK1 |  |  | 191 | 0.255 | 0.7168 | Yes |
Table: GSEA details [plain text format]

  

Fig 2: NB\_KI\_CLUSTER5: Random ES distribution      
 Gene set null distribution of ES for **NB\_KI\_CLUSTER5**
